# Immunophenotypic assessment of clonal plasma cells and B-cells in bone marrow and blood in the diagnostic classification of early stage monoclonal gammopathies: an iSTOPMM study

**DOI:** 10.1038/s41408-023-00944-1

**Published:** 2023-12-11

**Authors:** Oihane Pérez-Escurza, Juan Flores-Montero, Jón Þórir Óskarsson, Luzalba Sanoja-Flores, Julio del Pozo, Quentin Lecrevisse, Silvia Martín, Elín Ruth Reed, Guðlaug Katrín Hákonardóttir, Stephen Harding, Sigrún Þorsteinsdóttir, Sæmundur Rögnvaldsson, Thorvardur Jon Love, Brian Durie, Sigurður Yngvi Kristinsson, Alberto Orfao

**Affiliations:** 1 Translational and Clinical Research Program, Cancer Research Center (IBMCC, CSIC—University of Salamanca); Cytometry Service, NUCLEUS; Department of Medicine, University of Salamanca (Universidad de Salamanca), Salamanca, Spain; 2 Institute of Biomedical Research of Salamanca (IBSAL), Salamanca, Spain; 3 Department of Medicine, University of Salamanca (Universidad de Salamanca), Salamanca, Spain; 4 Biomedical Research Networking Centre Consortium of Oncology (CIBERONC), Instituto de Salud Carlos III, Madrid, Spain; 5 Department of Hematology, University Hospital of Salamanca, Salamanca, Spain; 6 Faculty of Medicine, University of Iceland, Reykjavík, Iceland; 7 Institute of Biomedicine of Seville, Department of Hematology, University Hospital Virgen del Rocío of the Consejo Superior de Investigaciones Científicas (CSIC), University of Seville, Seville, Spain; 8 The Binding Site Group, Birmingham, UK; 9 Rigshospitalet, Copenhagen, Denmark; 10 Department of Science, Landspitali University Hospital, Reykjavík, Iceland; 11 Department of Hematology and Oncology, Cedars-Sinai Medical Center, Los Angeles, CA USA

**Keywords:** Myeloma, Myeloma

## Abstract

Monoclonal gammopathy of undetermined significance (MGUS) is the earliest discernible stage of multiple myeloma (MM) and Waldenström’s macroglobulinemia (WM). Early diagnosis of MG may be compromised by the low-level infiltration, undetectable to low-sensitive methodologies. Here, we investigated the prevalence and immunophenotypic profile of clonal (c) plasma cells (PC) and/or cB-lymphocytes in bone marrow (BM) and blood of subjects with a serum M-component from the iSTOPMM program, using high-sensitive next-generation flow cytometry (NGF), and its utility in the diagnostic classification of early-stage MG. We studied 164 paired BM and blood samples from 82 subjects, focusing the analysis on: 55 MGUS, 12 smoldering MM (SMM) and 8 smoldering WM (SWM). cPC were detected in 84% of the BM samples and cB-lymphocytes in 45%, coexisting in 39% of cases. In 29% of patients, the phenotypic features of cPC and/or cB-lymphocytes allowed a more accurate disease classification, including: 19/55 (35%) MGUS, 1/12 (8%) SMM and 2/8 (25%) SWM. Blood samples were informative in 49% of the BM-positive cases. We demonstrated the utility of NGF for a more accurate diagnostic classification of early-stage MG.

## Introduction

Plasma cell (PC) neoplasms comprise a heterogeneous group of neoplastic diseases of long-lived clonal (c) PC that accumulate in bone marrow (BM), and to a lesser extent, also in other tissues [1, 2]. Expanded cPC might also circulate in blood and they usually secrete a monoclonal (M) immunoglobulin (Ig) (i.e., the M-component) of the IgM, IgG, or IgA isotype, and, less frequently, an Ig light chain (Ig-LC), that is detectable in serum and/or urine [1–3].

Among all PC neoplasms, monoclonal gammopathy of undetermined significance (MGUS) is by far the most prevalent (3–5% of adults >50 years) [3, 4]. It is defined by the presence of a smaller (<30 g/l) serum M-component and <10% BM infiltration by PC, with no signs of myeloma-related end-organ damage [3–7]. At present, it is well-established that IgA, IgG and Ig-LC MGUS may progress to smoldering multiple myeloma (SMM), which has a prevalence of 0.53% of adults >40 years [8], and (clinical/symptomatic) multiple myeloma (MM) at a rate of ≈1%/year, while SMM cases progress to MM at a higher rate of ≈10%/year [3, 9–11]. Progression to MM is characterized by an increase in the percentage of BM PC (i.e., >10%) and the serum M-component (≥30 g/l), together with the emergence of end-organ damage (e.g., CRAB for hypercalcemia, renal insufficiency, anemia and/or bone lesions) or occurrence of other myeloma defining events [3, 5–7, 12]. In contrast, IgM-MGUS rarely evolves to an IgM-MM, and more frequently progresses to a subclinical/smoldering Waldenström’s macroglobulinemia (SWM), and WM with a progressive increase in serum IgM M-protein (≥30 g/l) in association with ≥10% infiltration of BM by (clonal) lymphoplasmacytic cells and, in case of WM, also organomegalies [1, 2, 6, 9, 12–16]. Thus, in both instances, intermediate/smoldering forms of the disease (SMM or SWM) have been defined based on a yet subclinical disease, but higher levels of serum M-component (≥30 g/l), and/or ≥10% infiltration either by cPC (in the absence of myeloma defining events) or by lymphoplasmacytic cells (in the absence of organomegalies), respectively [3, 10, 11, 13, 15, 16]. Of note, it has been recently proposed that SMM patients with an 80% risk of progression at two years should also be considered as MM and offered treatment [10, 17].

In addition to typical MGUS cases, a serum M-component associated with the involvement of extramedullary tissues (e.g., bone, skin and/or lymph nodes) by cPC without or with lymphoplasmacytic cells, might also be found (with no or minimal BM involvement) in case of solitary (and multifocal) plasmacytomas and lymphoplasmacytic lymphomas, respectively [1, 2, 6, 18, 19], with variable rates of disease progression and need for systemic therapy. Because of all the above, a national screening program for MG -the Iceland Screens, Treats, or Prevents Multiple Myeloma (iSTOPMM) project- [20] has recently started in Iceland aimed at defining the potential utility of early diagnosis and therapeutic interventions in subjects from the general population who present with an MG in the absence of other signs of disease.

Independently of the specific disease subtype, the diagnosis and classification of PC neoplasms currently require a BM study. However, due to the low levels of infiltration in BM, particularly at the early phases of the disease, and the limited sensitivity and specificity of cytomorphology for the discrimination between normal/reactive and neoplastic PC and B-cells, these might go undetected in current routine diagnostic practice, at the same time coexistence of distinct unrelated populations of clonal B-cells and/or PC might lead to misdiagnosis [21].

In recent years, next-generation flow cytometry (NGF) has proven to be a high-sensitive technique for the detection of low cPC and clonal B-lymphocyte levels in BM and blood based on their uniquely distinct aberrant immunophenotype, even at the very early stages of disease, when they are present at very low frequencies of e.g. 10^−5^– 10^−6^ [22–28]. In addition, cPC from SMM/MM and SWM/WM has long been shown to display clearly different immunophenotypic features, which mimic those of normal/reactive BM PC and blood circulating plasmablasts and/or related lymphoplasmacytic lymphoma-like B-cells, respectively [29–31].

Here, we investigated the presence and immunophenotypic characteristics of cPC and cB-lymphocytes in paired BM and blood samples from a series of (otherwise) healthy adults from Iceland who had been screened for the presence of a serum M-component and an underlying monoclonal gammopathy (MG) within the iSTOPMM program [20]. Our main goal was to gain insight into the potential utility of high-sensitive NGF for the identification and characterization of cPC and cB-lymphocytes in BM and blood of these individuals, for more accurate diagnosis and classification of patients presenting with MG, particularly at the (very) early stages of disease.

## Materials and methods

### Patients and samples

A total of 82 (otherwise) healthy adults aged ≥40 years (44 men and 38 women; median age of 67 years, ranging from 45 to 86 years) with a positive screening for a serum M-component by high-sensitive capillary zone electrophoresis and/or an altered free-Ig light chain (FLC)-ratio (The Binding Site Group Ltd, Birmingham, UK) [20] from the iSTOPMM project who were referred for a confirmatory serum M-component and underwent a BM aspiration procedure in a follow-up visit, fulfilling the diagnostic criteria for SWM/lymphoplasmacytic lymphoma, MGUS or SMM according to the International Myeloma Working Group (IMWG) [3, 5, 6], were selected to be further enrolled in this study. Subjects with prior diagnosis of lymphoproliferative disorders or with mature B cell/plasma cell neoplasms other than SWM, MGUS, and SMM, were excluded from this study. Following the IMWG diagnostic criteria [3, 5, 6], 82 subjects were initially enrolled in the study and subsequently classified into the following disease categories: i) MGUS, 55 subjects; ii) SMM, 12 patients; iii) MM, one case; iv) SWM, 8 donors; v) plasmacytoma, one case; and vi) one chronic lymphocytic leukemia (CLL) subject. In the remaining 4 individuals, the presence of a serum M-component was not confirmed in subsequent evaluations performed after the initial screening and were therefore classified as transient M-component cases [32]. Sample analysis was performed blindly from the distribution of subjects by disease category groups. Based on the predefined exclusion criteria, 7/82 cases (one diagnosed with symptomatic MM, one with plasmacytoma, one with CLL and 4 with a transient M-component) were further excluded from subsequent analyses. The remaining 75/82 subjects (MGUS, SMM, and SWM), represented the core series included in the analyses performed in this study.

A total of 150 EDTA-anticoagulated (paired) BM aspirated samples (2 ml/sample; *n* = 75) and blood samples (10 ml/sample; *n* = 75) were collected in Iceland from the 75 individuals enrolled in this study and sent by overnight express courier to the University of Salamanca (Salamanca, Spain) for processing within 36 h after collection.

All subjects enrolled in this study provided their informed consent to participate according to the Declaration of Helsinki. The study met the ethics criteria of Iceland and Spain and was approved by the Icelandic National Bioethics Committee (NCT03327597) as well as by the Icelandic Data Protection Agency, permission being granted from the Spanish Ministry of Health for the transfer of all biological samples for this study (Registration number: 2020/252).

### Immunophenotypic studies

Paired BM (1 ml) and blood (4 ml) samples from each subject were stained in parallel with the EuroFlow NGF-MM minimal residual disease (MRD) (2-tube, 8-color) antibody (Ab) panel [26] (Supplementary Table 1, Panel A) and the Lymphoid Screening Tube [28] (Supplementary Table 1, Panel B), using the BD OneFlow (BD, Becton/Dickinson Bioscience, San Jose, CA, USA) dried tubes [33] as per the EuroFlow standard operating procedures (SOPs) for sample preparation and staining available at www.euroflow.org [26, 34]. Stained samples were acquired in FACSCanto^TM^ II (BD) or FACSLyric^TM^ (BD) flow cytometers for a median number of 10^7^ (2–15 × 10^6^) and 1.3 × 10^6^ (0.2–5 × 10^6^) cells, respectively. For (automated) flow cytometry data analysis, the Infinicyt^TM^ software (version 2.0.5.d; Cytognos SL., Salamanca, Spain) was used. Following previous criteria [26], the limit of detection for each cell population was set at ≥20 cells.

Clonal PC and cB-lymphocytes were identified based on their unique aberrant phenotypes and/or Ig-LC restriction [26, 27]. Flow cytometry-based immunophenotypic criteria were subsequently applied for further phenotypic classification of the clonal/aberrant PC and B-lymphocytes [13, 35, 36], which included: i) the presence of cPC and/or cB-cells in BM and; ii) the specific immunophenotypic characteristics of such cPC and cB-lymphocytes, respectively [13, 35, 36].

### Statistical methods

The Spearman’s correlation coefficient (Spearman’s Rho) was used to calculate the degree of correlation between two (quantitative) variables. The Fisher’s exact test and, either the Mann-Whitney *U* or the Kruskal-Wallis tests, were used to assess the statistical significance of differences observed between groups of individuals for categorical and continuous variables, respectively. In turn, the MacNemar test was employed for the comparison of paired BM vs blood data. Bilateral significance was determined in these tests. Receiver operating characteristic (ROC) curve analysis was used to calculate the area under the curve defined by the percentage of cPC with respect to total BM PC that best distinguished between different disease groups. For all statistical analysis, the SPSS IBM-Statistical software Package for Social Sciences (SPSS v25.0; IBM Corp, Armonk, NY, USA) was used. Statistical significance was set at *p* values < 0.05.

## Results

### Serum M-component isotype and concentration

A persistent serum M-component and/or Ig-LC was detected in all (75/75, 100%) of the subjects investigated. IgG was the most frequently detected M-component (31/75, 41%), followed by IgM (26/75, 35%), IgA (14/75, 19%) and Ig-LC (3/75, 4%). One (1/75, 1%) case showed two different (IgG/κ and IgM/κ) M-peaks. The distribution of the different serum M-components per diagnostic category was as follows: MGUS, 24/55 subjects (43%) had an IgG M-component, 18/55 (33%) were IgM, 11/55 (20%) IgA and 2/55 (4%) LC cases; SMM, 7/12 (58%) IgG, 3/12 (25%) IgA, 1/12 (8%) LC and 1/12 (8%) IgG plus IgM subjects; and, SWM, 8/8 (100%) IgM cases per definition (Supplementary Table 2). Median (range) concentration of the M-component in serum increased from 0.8 g/l (<0.1–8.8 g/l) in MGUS to 2.5 g/l (0.4–17.2 g/l) in SWM and 7.1 g/l (<0.1–15.8 g/l) in SMM.

### Prevalence of clonal PC and/or clonal B-lymphocytes in healthy adults presenting with a serum M-component

Overall, cPC and/or cB-lymphocytes were detected in BM and/or blood of 68/75 (91%) individuals investigated. These included 49/55 MGUS (89%) subjects, 12/12 SMM patients (100%) and 7/8 SWM subjects (88%) (Table 1). As detailed in Table 1, cPC were found in the great majority (63/75, 84%) of MG cases, while cB-lymphocytes were detected in less than half of the same individuals (34/75, 45%). In 34/75 (45%) subjects only cPC in the absence of cB-lymphocytes were found, while 5/75 (7%) cases displayed cB-lymphocytes without cPC, both populations of cPC and cB-lymphocytes coexisting in the remaining 29/75 (39%) subjects. For a detailed description of the frequency and type of Ig on each diagnostic category and their distribution according to the clonal cell type detected, see Supplementary Table 2. Among MGUS cases, cPC were detected in most (44/55, 80%) patients, either alone (27/55, 49%) or together with cB-lymphocytes (17/55, 31%). From the remaining MGUS subjects, 5/55 (9%) showed only cB-lymphocytes, while no clonal PC nor cB-lymphocytes were found in the other 6/55 (11%). In turn, 12/12 SMM patients showed cPC either in the absence (7/12, 58%) or the presence (5/12, 42%) of coexisting cB-lymphocytes. Finally, the coexistence of cPC and cB-lymphocytes was confirmed in all but one SWM patients (7/8, 88%), in whom no cPC or cB-lymphocytes were detected.

**Table 1:** Frequency of clonal plasma cells (cPC) and clonal B-lymphocytes (cB-cells) in BM of included subjects presenting with an M-component in serum distributed according to diagnosis as per the IMWG criteria (*n* = 75/82).

Diagnosis	Clonal B-cell type			*p* value	Total
	cPC only	cPC + cB-cells	cB-cells only		
MGUS (*n* = 55)	27 (49%)^^^	17 (31%)^^^	5 (9%)^*!^	*<0.001*	49 (89%)
SMM (*n* = 12)	7 (58%)^^^	5 (42%)	0 (0%)^*!^	*0.008*	12 (100%)
SWM (*n* = 8)	0 (0%)	7 (88%)^*^	0 (0%)^!^	*<0.001*	7 (88%)
*p* value	*0.02*	*0.009*	*0.38*		*0.47*
Total (*n* = 75)	34/75 (45%)	29/75 (39%)	5/75 (7%)^*!^	*<0.001*	68/75 (91%)

*IMWG* International Myeloma Working Group, *MGUS* monoclonal gammopathy of undetermined significance, *SMM* smoldering multiple myeloma, *SWM* smoldering Waldenström’s macroglobulinemia.

^^^*p* < 0.05 vs SWM; ^*^p < 0.05 vs cPC; ^!^*p* < 0.05 vs cPC+cB-cells.

Interestingly, in half of those individuals harboring both cPC and cB-lymphocytes (15/29, 52%), both (clonal) cell populations displayed closely related phenotypic features, including the same Ig light chain. These mostly consisted of SWM (6/7, 86%) and MGUS cases (8/17, 47%), in addition to 1/5 (20%) SMM patients (Table 2). Among those 14/29 (48%) cases with coexisting but phenotypically unrelated cPC and cB-lymphocytes, the later cells showed immunophenotypic features of CLL-like MBL in 4/4 (100%) SMM patients and in 7/10 (70%) MGUS cases, non-CLL-like MBL clones being found in the other 3/10 (30%) MGUS individuals.

**Table 2:** Phenotypically related clonal plasma cells (cPC) and clonal B-lymphocytes (cB-cells) coexisting in included subjects presenting with a serum M-component distributed according to diagnosis as per the IMWG criteria (*n* = 29).

Diagnosis	Related cPC + cB-cells
MGUS (*n* = 17)	8 (47%)
SMM (*n* = 5)	1 (20%)
SWM (*n* = 7)	6 (86%)^^^
*p* value	*<0.001*
Total (*n* = 29)	15/29 (52%)

^^^*p* < 0.05 vs other groups.

*IMWG* International Myeloma Working Group, *MGUS* monoclonal gammopathy of undetermined significance, *SMM* smoldering multiple myeloma, *SWM* smoldering Waldenström’s macroglobulinemia.

### Utility of immunophenotyping for the diagnostic classification of patients with a serum M-component

Detailed analysis of the phenotypic features of cPC and cB-lymphocytes found in patients classified into the different diagnostic subtypes of PC neoplasms by the IMWG criteria [6] showed discrepancies in 22/75 (29%) cases. These included a significant fraction of MGUS (19/55, 35%), together with fewer -2/8 (25%)- SWM cases, and one SMM -1/12 (8%)- patient (Table 3).

**Table 3:** Immunophenotypic profile of clonal plasma cells (cPC) and clonal B-lymphocytes (cB-cells) identified in 22 patients presenting with a discrepant immunophenotypic profile of cB-cells and/or cPC as compared to the established diagnosis according to the IMWG criteria [6].

Diagnosis	Serum M-component	N. of discrepant cases^*^ (*n* = 22)	Immunophenotypic profile of clonal PC and B-cells				*p* value
			No cPC or cB-cells detected (*n* = 7)	MM-like cPC (*n* = 34)	WM-like cB-cells and cPC (*n* = 15)	MBL-like cB-cells (*n* = 5)	
**MGUS** (*n* = 55)		**19/55 (35%)**					
	**IgM** (*n* = 18)	16/18 (89%)	4 (25%)	0 (0%)^!^	8 (50%)	4 (25%)	*0.01*
	**IgG** (*n* = 24)	2/24 (8%)	1 (50%)	0 (0%)	0 (0%)	1 (50%)	*0.45*
	**Light-chain** (*n* = 2)	1/2 (50%)	1 (100%)	0 (0%)	0 (0%)	0 (0%)	*0.26*
**SMM** (*n* = 12)		**1/12 (8%)**					
	**IgG** + **IgM** (*n* = 1)	1/1 (100%)	0 (0%)	0 (0%)	1 (100%)	0 (0%)	*0.26*
**SWM** (*n* = 8)		**2/8 (25%)**					
	**IgM** (*n* = 8)	2/8 (25%)	1 (50%)	1 (50%)^^^	0 (0%)	0 (0%)	*0.45*
***p*-value**		***0.44***					
**Total** (*n* = 75)		**22/75 (29%)**	**7/7 (100%)**^**’**^	**1/34 (3%)**^**!**^	**9/15 (60%)**	**5/5 (100%)**	***0.04***

*BM* bone marrow, *cB-cells* clonal B-lymphocytes, *cPC* clonal plasma cells, *IMWG* International Myeloma Working Group, *MGUS* monoclonal gammopathy of undetermined significance, *MBL* monoclonal B-cell lymphocytosis, *MM* clinical/symptomatic multiple myeloma, *PC* plasma cell, *SMM* smoldering multiple myeloma, *SWM* smoldering Waldenström’s macroglobulinemia, *WM* Waldenström’s macroglobulinemia.

*Based on the presence and the immunophenotypic characteristics of cPC and/or cB-cells in BM [35]; ^^^this sample showed additional MBL-like cB-lymphocytes; ^!^*p* < 0.05 vs WM-like cB-lymphocytes and cPC; ’*p* < 0.05 vs MM-like cPC.

In bold, the total number of cases per disease category and per population(s) of clonal cells detected.

Among the 19 discrepant MGUS cases, most corresponded to IgM-MGUS (16/19, 84%), another 2/19 (11%) were IgG-MGUS and the remaining patient had been classified as LC-MGUS (1/19, 5%) (Table 3, Supplementary Table 3). Interestingly, in half of the discrepant IgM-MGUS cases, cPC coexisted with cB-lymphocytes with a WM-like immunophenotype (8/16, 50%), while the other discrepant MGUS cases (11/19, 58%) included 8/16 IgM-MGUS in whom cPC could not be detected in BM nor in blood, either in the absence of other cB-cells (4/8, 50%) or in the presence of CLL-like or non CLL-like MBL cB-lymphocytes (4/8, 50%). The remaining 3 discrepant MGUS cases corresponded to 2 subjects (one IgG and one Ig-LC) without evidence of clonal cells, and one IgG showing only cB-cells. Among SWM patients, the 2/8 discrepant cases were due to the lack of cPC and cB-lymphocytes as detected by NGF in one case, and the coexistence of IgM cPC with a typical MM-like phenotype and an unrelated CLL-like MBL cB-cell population suggesting independent disorders. Finally, the (single) SMM discrepant case showed co-existence in serum of two IgG/κ and IgM/κ M-components, associated with phenotypically related cPC and cB-lymphocytes, both expressing Igκ, with an WM-like phenotype.

### Presence of clonal PC and/or B-lymphocytes in BM vs blood

**Fig. 1:**
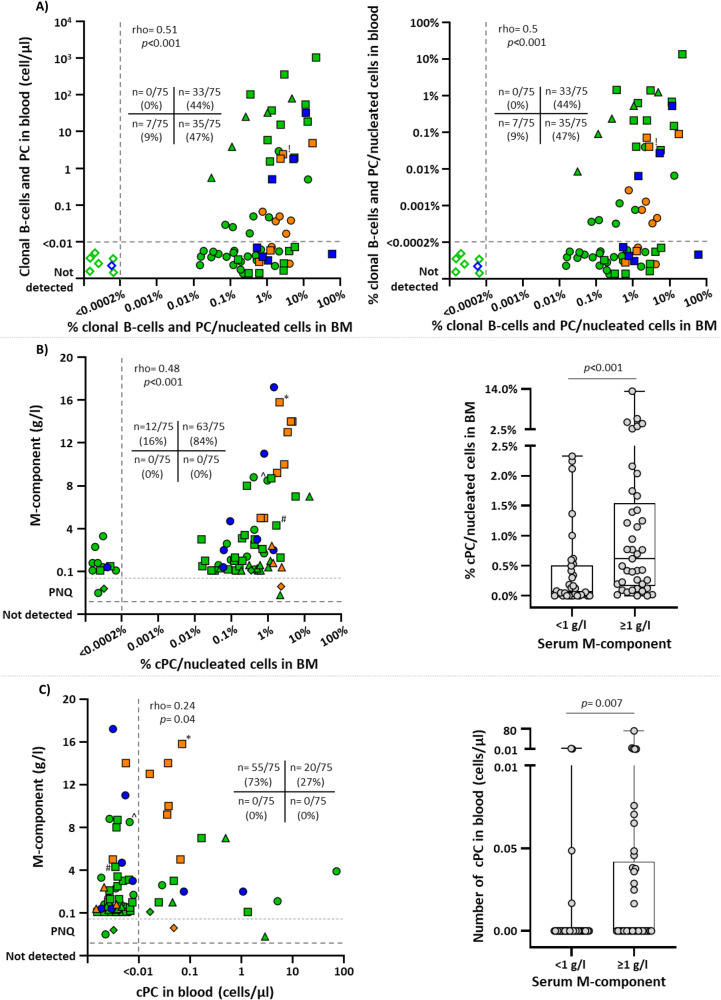
Relationship between the number of cPC and cB-lymphocytes in BM and blood and the concentration of serum M-component in individual subjects. In (**A**), the correlation between the number of clonal plasma cells and clonal B-lymphocytes in BM (percentage values) and blood (number of cells/µl and percentage values) observed for each color-coded individual according to diagnosis is shown. **B** and **C** show the correlation between the concentration of serum M-component and the number of clonal plasma cells in bone marrow and in blood of the same individuals, respectively. In all plots, MGUS patients are depicted in green, SMM cases are colored in orange and SWM in blue. In (**A**), the type of clonal cell detected is distinguished by shapes: circles, cPC only; squares, coexisting cPC and cB-lymphocytes; triangles, cB-lymphocytes only; and empty rhombs, no clonal cells. In turn, in (**B** and **C**) (left plots) distinct shapes denote the type of serum M-component: circles, IgM+; squares, IgG+; triangles, IgA+; rhombs, light chain; and empty rhombs, no paraprotein. Box plots in (**B**, **C**) (right plots) show median values (middle line), the 25th and 75th percentile values (boxes), minimum and maximum values (whiskers) and the percentages of cPC in BM for each individual case (inner circles). BM bone marrow, cB-cells clonal B-lymphocytes, cPC clonal plasma cells, MGUS monoclonal gammopathy of undetermined significance, PNQ positive not quantifiable, SMM smoldering multiple myeloma, SWM smoldering Waldenström’s macroglobulinemia. ^!^Detection of two cPC and one cB-cell populations in blood and one cPC and the cB-cell populations in BM; ^*^Two M-peaks detected by immunofixation, IgG and IgM; ^^^Two M-peaks detected by immunofixation, IgM and IgM; ^#^Two M-peaks detected by immunofixation, IgG and IgG.

Overall, circulating cPC and/or cB-lymphocytes were found in 33/75 (44%) cases investigated, including half (33/68, 49%) of those cases who carried cPC and/or cB-lymphocytes in BM (Fig. 1A, Supplementary Table 4). These consisted of 22/55 (40%) MGUS -22/49 (45%) MGUS positive cases in BM-, 8/12 (67%) SMM, and 3/8 (38%) SWM -3/7 (43%) SWM positive subjects in BM- (Fig. 1A, Supplementary Table 4). Briefly, circulating cPC were detected in blood of 12/34 (35%) cases showing cPC (only) in BM -5/12 (42%) SMM and 7/55 (13%) MGUS cases- (Fig. 1A, Supplementary Table 4). Among those individuals who showed both cPC and cB-lymphocytes in BM (29/75, 39%), both populations were also detected in blood in 8/29 (28%) cases -2/8 (25%) SWM, 2/12 (17%) SMM and 4/55 (7%) MGUS cases- (Fig. 1A, Supplementary Table 4). In the remaining cases with coexisting cPC and cB-lymphocytes in BM (21/29, 72%) no circulating cPC populations were found. However, in 8/21 (38%) of these later subjects, including 6/8 (75%) MGUS, 1/8 (12%) SWM and 1/8 (12%) SMM cases, clonal (CLL-like or non-CLL-like) MBL populations were identified; the other 13/21 (62%) cases showed neither cPC nor cB-lymphocytes circulating in blood and they included 7/13 (54%) MGUS, 4/13 (31%) SWM and 2/13 (15%) SMM (Fig. 1A, Supplementary Table 4). Finally, in all cases in which a cB-lymphocyte population had been detected in BM in the absence of cPC (5/75, 7%), these were also present in blood (5/5, 100%) in the MGUS patients (Fig. 1A, Supplementary Table 4). Interestingly, in one SMM case with an IgG serum M-component, two different cPC (Igκ and Igλ, with otherwise overlapping phenotypic characteristics) together with a CLL-like MBL clone were detected in blood, while a single cPC population (Igλ) together with a CLL-like MBL was found in BM (Fig. 1A).

Overall, the number of circulating cPC and/or cB-lymphocytes in blood significantly correlated (rho = 0.51; *p* < 0.001) with the percentage of clonal cells (cPC plus cB-lymphocytes) in BM (Fig. 1A), particularly among cases who had only cB-lymphocytes in the absence of cPC (rho = 1; *p* < 0.001). Of note, progressively higher (median; range) percentages of BM clonal cells were found in MGUS (0.34%; <0.0002–22.2%), SWM (1.2%; <0.0002–61%) (*p* = 0.08 vs MGUS) and SMM (2.3%; 0.6–17.8%) patients (*p* = 0.003 vs MGUS), while no significant differences were found in the median (range) distribution of clonal cells (cPC or cB-lymphocytes) in blood between MGUS (<0.01 cells/µl; <0.01–1 053 cells/µl), SWM (<0.01 cells/µl; <0.01–32.2 cells/µl) and SMM (0.04 cells/µl; <0.01–4.8 cells/µl) (*p* = 0.57).

Furthermore, in 13 (17%) cases with cPC, and in 12 (16%) cases with cB-cells, BM analysis revealed the presence of ≥2 unrelated PC and/or B-cells clones, while this was also found in blood in a minority -1/13 (8%) and 4/12 (33%), respectively- of those same cases.

### Correlation between the serum M-component and the number of clonal PC in blood and BM

The number of cPC in blood (rho = 0.24; *p* = 0.04) and, particularly in BM (rho = 0.48; *p* < 0.001) (MGUS, rho = 0.37, *p* = 0.005; SMM, rho = 0.47, *p* = 0.12; and SWM, rho = 0.78, *p* = 0.02), significantly correlated with the concentration of the serum M-component. In line with these findings, significantly higher numbers of cPC in BM (*p* < 0.001) and in blood (*p* = 0.007) were found in cases presenting with <1 g/l vs ≥1 g/l of M-component in serum (Fig. 1B, C).

### Association between the percentage of cPC/total PC in BM and B-cell counts in blood in the distinct diagnostic subtypes of MG

**Fig. 2:**
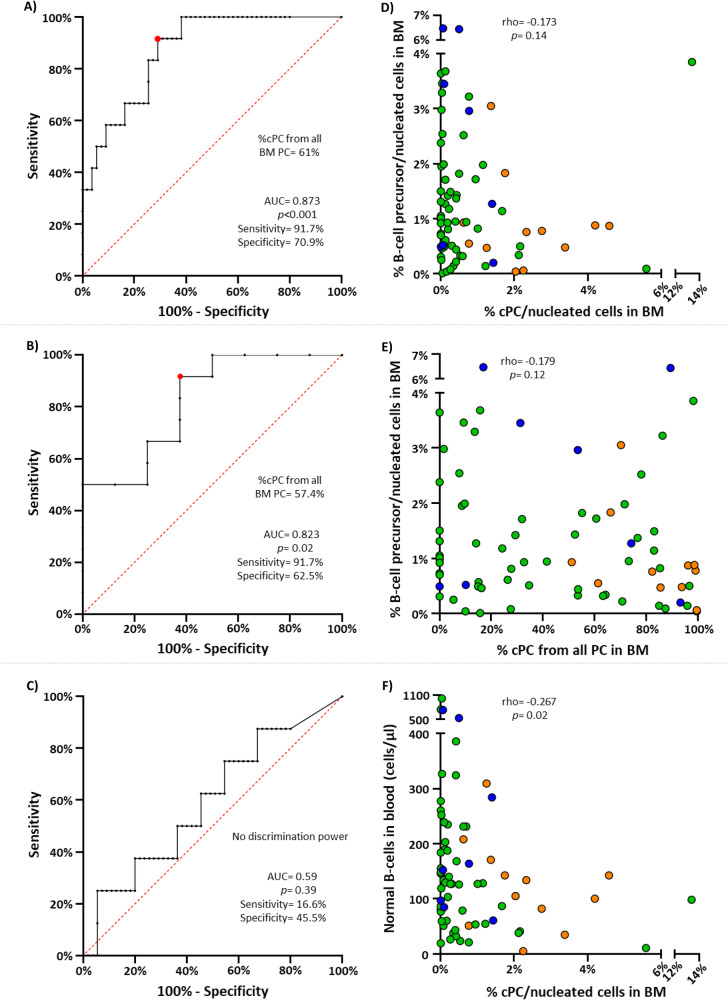
Impact of the percentages of cPC in BM on disease classification and normal B-cell production. In (**A**–**C**) receiver operating characteristic (ROC) curve plots showing the power of percentage values of cPC/from all BM PC to discriminate between MGUS and SMM (**A**; 61%), SMM and SWM (**B**; 57.4%) and between MGUS and SWM (**C**; no discrimination power), are shown. In (**D**–**F**) the correlation between the percentage of B-cell precursors in BM vs the percentage of cPC from all BM nucleated cells (**D**) and BM PC (**B**) are shown, while in (**F**) the correlation between number of normal circulating B-lymphocytes in blood and the percentage of cPC from all BM nucleated cells is displayed. In Panels D-F, MGUS patients are depicted in green, SMM cases in orange and SWM in blue. AUC area under the curve, BM bone marrow, cPC clonal plasma cell, MGUS monoclonal gammopathy of undetermined significance, PC plasma cell, SMM smoldering multiple myeloma, SWM smoldering Waldenström’s macroglobulinemia.

Overall, a significantly higher (median; range) percentage of cPC/total PC in BM was observed in SMM (89.6%; 51.2–99.5%) compared to MGUS (27.6%; 0–98.2%) (*p* < 0.001) and to a lesser extent also, SWM cases (42.4%; 0–93.2%) (*p* = 0.03). ROC curve analysis showed that the presence of >60% cPC from all BM PC, was the most accurate cut-off value to discriminate between MGUS and SMM -area under the curve (AUC) of 0.873; *p* < 0.001- with a sensitivity of 91.7% and a specificity of 70.9% (Fig. 2A). Similarly, the presence of 57.4% cPC/total BM PC also allowed discrimination between SMM and SWM (AUC of 0.823; *p* = 0.02) with a similar sensitivity (91.7%) and specificity (62.5%) (Fig. 2B). In contrast, the percentage of cPC/total BM PC was similar in MGUS and SWM and did not allow clearcut distinction between both diagnostic entities (*p* = 0.39) (Fig. 2C).

Importantly, at the early stages of MG (MGUS and SMM) a tendency towards an inverse correlation was observed between the percentage of cPC -both out of nucleated cells (rho = -0.173; *p* = 0.14) and within the total PC population (rho = -0.179; *p* = 0.12)- and the relative distribution of other B-cell population compartments (e.g., B-cell precursors) in BM (Fig. 2D, E respectively), such inverse correlation being more pronounced in SMM patients (rho = -0.566; *p* = 0.06). In line with these findings, a significant (inverse) correlation was also found between the percentage of cPC in BM and the number of normal mature B-lymphocytes circulating in blood (rho = -0.267; *p* = 0.02) (Fig. 2F), even when MGUS and SMM subjects were separately considered -(rho = -0.351; *p* = 0.009) and (rho = -0.441; *p* = 0.15), respectively.

## Discussion

MM and WM are malignant hematological neoplasms characterized by the presence of a serum M-component secreted by an expanded population of cPC, which are preceded by an MGUS, followed by intermediate SMM or SWM stages [1, 2]. In recent years, several risk factors have been identified which are associated with a greater risk of progression from MGUS, SMM or SWM to MM and WM, respectively. Preliminary data on (ultrahigh-risk) SMM and, to a lesser extent, also MGUS patients, but not SWM [16, 37], support a potential benefit for early therapeutic interventions [10, 17, 20]. These observations have highlighted the potential utility of screening for the presence of a serum M-component in the general population. However, at these early stages of PC neoplasms, low levels of BM infiltration by clonal PC and/or B-cells are detected (in association with smaller serum M-component) which might go undetected by conventional cytological and histopathological approaches [26, 38, 39]. Such limitation might be overcome through the use of NGF techniques because of their higher sensitivity for the identification and immunophenotypic characterization of minimal numbers of cPC and cB-lymphocytes both in BM and blood [26, 40]. Here, we used NGF to investigate its potential utility in confirming the presence of neoplastic B-cells in healthy subjects and patients presenting with a serum M-component in a routine laboratory screening test, and its contribution to a more accurate classification of the underlying PC disorder independently of the isotype of the secreted M-protein.

Overall, the concentration (median) of the M-component found in MGUS cases in our cohort was significantly lower than that observed in previous series reported in the literature, based on both hospital-referred patients (5–12 g/l) [41–43] and other population-based studies (3.9 g/l) [44], which probably reflects the diagnostic screening strategy carried out within the study [20], potentially translated also in a higher frequency of cases adjusted per age.

Despite the lower serum M-component levels found in our versus the previously reported cohorts [41–44], NGF demonstrated the presence of cPC cells in the BM of 84% of cases with a persisting serum M-component associated with a population of clonal B-lymphocytes in nearly half (46%) of these cases. Of note, both clonal cell populations coexisted in more than a third of these individuals, among whom they shared immunophenotypic features consistent with related conditions in half of the cases. As expected, virtually all SMM cases showed the presence of cPC in BM in the absence of related cB-lymphocytes [1–3, 5–7], coexistence of cB-lymphocytes systematically corresponding to CLL-like MBL, except for one individual showing SWM-like features associated with two M-peaks (a major IgGκ-peak and a minor IgMκ-peak) [1, 2, 13, 15, 16]. In contrast, almost every SWM case showed coexistence of two related populations of cPC and cB-lymphocytes, with an immunophenotypic profile consistent with a diagnosis of SWM in all but one patient who had cPC consistent with SMM/MM coexisting with a CLL-like MBL [1, 2, 13, 15, 16]. These results support the diagnosis of an IgM (MM-like) MGUS in the later subject, in contrast to the other IgM-SWM cases, pointing out the utility of NGF for the (currently challenging) differential diagnosis between IgM-MGUS and other IgM-related neoplasms (e.g., SWM) [45–47]. In contrast, the immunophenotypic profiles found in MGUS cases were significantly more heterogeneous, typically consisting of a cPC population alone or together with either a coexisting CLL-like or a non-CLL-like MBL population in two-thirds of the cases. In the remaining MGUS cases, no cPC were detected or two related populations of cPC and cB-lymphocytes sharing SWM-like immunophenotypic profiles associated with the presence of an IgM-peak in the serum, were identified [1, 2, 13, 15, 16]. Overall, this later case plus two other IgM-MGUS cases found to display MM-like immunophenotypic profiles in their cPC, suggest a relatively higher incidence (4% vs 0.5–1% of MG) of IgM-MM-like MGUS cases in our vs previous cohorts [45–47]. Further detailed (e.g., genetic) characterization of cPC from these patients is required for their accurate diagnostic classification (via e.g., demonstration of the presence vs absence of the *MYD88*^*L265P*^ mutation in MM-like IgM-MGUS vs SWM) [45–47]. Of note, in around half of the other MGUS cases who showed no cPC in the BM, cB-lymphocytes with a CLL-like phenotype or, to a lesser extent, non-CLL-like MBL features, were detected; whether in the non-CLL-like MBL cases the clonal B-lymphocytes are responsible for the serum M-component remains to be established. Interestingly, however, in both the MGUS and the SWM cases in whom no cPC nor cB-lymphocytes were detected the median serum concentration of the M-component was significantly lower than that of the other MGUS and SWM cases, supporting the notion that these might correspond to the earliest stages of disease in which cPC in BM are below the detection limit of the NGF techniques. In line with this hypothesis, a significant correlation was observed in our cohort between the number of cPC (and cPC plus cB-cells) in BM or blood and the serum concentration of the M-peak, similarly to what has been previously reported in the literature [48–50]. Alternatively, the putative M-component found in these cases might also include a fraction of false positive cases, in whom longer follow-up studies are needed to confirm the reactive vs neoplastic nature of the M-peak. Interestingly, here we also confirm previous observations in hospital-based MGUS and SMM patients that the percentage of cPC in the BM by NGF clearly differs between MGUS and SWM on one side, and SMM on the other side, which reflects the different rates of expansion and accumulation of pathological cPC in BM throughout the whole spectrum of these diseases. Noteworthy, MBL (mostly CLL-like MBL) was detected in a significant fraction of our cases, its frequency being higher among individuals with (vs without) a coexisting cPC population.

Previous studies have shown the presence of circulating cPC by NGF in blood of a high percentage of SMM and MM, and to a less extent also MGUS cases [51–54], which could be used at diagnosis for a more comprehensive management of these patients, as regards the timing to perform a BM study. Thus, an additional goal of our study was to investigate the presence of circulating cPC in the blood of our subjects, in parallel to the BM study. Overall, in more than a third of those cases who showed cPC in BM, a cPC population with an identical immunophenotype was also found in blood. In addition, the majority of cases with coexisting cPC and cB-cells and all of those who had only clonal mature B-lymphocytes in BM, showed circulating cB-lymphocytes in blood as well. Despite previous reports from our group and others [51, 55] have found higher rates of circulating cPC in MGUS and SMM as compared to our current data, the lower rate reported here might be due to the differences in the nature of the distinct patient cohorts, since our cohort is based on a screening performed in the general population whereas the others correspond to hospital-based series [56, 57]. In line with this hypothesis, a significant correlation was found here between the percentage of clonal B-cells and cPC in BM and both their percentage and absolute number in blood, as well as between the percentage of cPC in blood and the serum concentration of the M-component [1–3, 58].

Most interestingly, here we found an inverse correlation between the percentage of cPC in the BM and both the percentage of BM B-cell precursors and the blood B-lymphocyte counts, supporting the notion that progressive accumulation of cPC in the BM of MG patients not only replaces normal PC in their BM niches, but it also progressively affects normal B-cell production, already at very early stages of disease [51, 59].

In summary, our results demonstrate that NGF immunophenotypic analysis of BM and, to a lesser extent also, blood samples from individuals at the very early stages of MG, provides complementary data to that obtained through conventional diagnostic procedures via a more precise definition of the clonal nature of the (either related or unrelated) cell populations involved, and contributes to a more accurate diagnosis and classification of PC neoplasms in around one third of MG presenting with a serum M-component in screening programs like iSTOPMM, being particularly useful in distinguishing SWM from IgM-MGUS cases. Based on these results, we envisage that high-sensitive blood investigation of circulating cPC and cB-cells might be included in the initial diagnostic workup of MG, to support disease diagnosis and classification and to help optimizing the timing of BM evaluation. The relative simplicity, high sensitivity, short time to results and worldwide availability of NGF, further facilitate the incorporation of the proposed assay in routine diagnostics for the characterization of patients with a serum M-component.

### Supplementary information

Supplementary tables

## Data Availability

The datasets generated during and/or analyzed during the current study are available from the corresponding author upon reasonable request.
