# Supplementary material for: Immunophenotypic assessment of clonal plasma cells and B-cells in bone marrow and blood in the diagnostic classification of early stage monoclonal gammopathies: an iSTOPMM study
Source: Blood Cancer J. 2023 Dec 11;13(1):182. doi: 10.1038/s41408-023-00944-1 (PMC10711003; doi:10.1038/s41408-023-00944-1)
Supplement: Supplementary file 1 — Supplementary tables [file 41408_2023_944_MOESM1_ESM.docx]

**Supplementary Table 1**. EuroFlow NGF multiple myeloma minimal residual disease (MM-MRD) antibody panel (A) and lymphocyte screening tube (LST; panel B) antibody combination used in this study.

| **Fluorochrome conjugate** | | | | | | | | | |
| --- | --- | --- | --- | --- | --- | --- | --- | --- | --- |
|  |  | **BV421** | **BV510** | **FITC** | **PE** | **PerCP Cy5.5** | **PE Cy7** | **APC** | **APC C750** |
| **Tube 1** |  | CD138 | CD27 | CD38 | CD56 | CD45 | CD19 | CD117 | CD81 |
| **Tube 2** |  | CD138 | CD27 | CD38 | CD56 | CD45 | CD19 | CyIgκ | CyIgλ |
|  | **Clone** | MI15 | O323 | Multiepitope | C5.9 | EO1 | SA287 | 104D2 Polyclonal | M38 Polyclonal |
|  | **Source** | BD | BioLegend | Cytognos | Cytognos | Cytognos | Cytognos | Cytognos | Cytognos |

PANEL A (MM-MRD panel):

PANEL B (BD OneFlow LST^TM^ dried tube):

| **Fluorochrome conjugate** | | | | | | | | |
| --- | --- | --- | --- | --- | --- | --- | --- | --- |
|  | **HV450** | **HV500c** | **FITC** | **PE** | **PerCP Cy5.5** | **PE Cy7** | **APC** | **APC-H7** |
| **Marker** | CD4 + CD20 | CD45 | CD8 + SmIgλ | CD56 + SmIgκ | CD5 | CD19 + TCRγδ | SmCD3 | CD38 |
| **Clone** | SK3 + L279 | 2D1 | SK1 + 1-155-2 | MY31 + TB28-2 | L17F12 | SJ25-C1 + 11F2 | SK7 | HB7 |

APC, allophycocyanine; APC-C750, allophycocyanine-C750; APCH7, allophycocyanine-hilite®7; BD, Becton Dickinson; BV, brilliant violet; Cy, cytoplasmic; FITC, fluorescein isothiocyanate; HV, horizon violet dye; LST, lymphoid screening tube; MM-MRD, multiple myeloma minimal residual disease; NGF, next generation flow; PE, phycoerythrin; PE-Cy7, phycoerythrin-cyanine7; PerCP Cy5.5, peridinin chlorophyll protein-cyanine5.5; Sm, surface membrane.

**Supplementary Table 2**. Frequency of clonal plasma cells (cPC) and clonal B-lymphocytes (cB-cells) detected in bone marrow vs blood of included subjects presenting with an M-component in serum distributed according to diagnosis as per the IMWG criteria and the specific heavy chain involved.

| **Diagnosis** | **Heavy chain isotype** | **Clonal B-cell type** | | | | **Total** |
| --- | --- | --- | --- | --- | --- | --- |
|  |  | **Blood** | **BM** | | |  |
|  |  |  | **cPC only**  (n=34) | **cPC+cB-cells** (n= 29) | **cB-cells only**  (n= 5) |  |
| **MGUS**  (n= 55) | **IgM+** 18/55 (33%) |  | **2 (11%)** | **8 (44%)** | **4 (22%)** | **14 (78%)** |
|  |  | None | 1 (6%) | 4 (22%) | 0 (0%) | 5 (28%) |
|  |  | cPC only | 1 (6%) | 0 (0%) | 0 (0%) | 1 (6%) |
|  |  | cPC+cB-cells | 0 (0%) | 2 (11%) | 0 (0%) | 2 (11%) |
|  |  | cB-cells only | 0 (0%) | 2 (11%) | 4 (22%) | 6 (33%) |
|  |  | Subtotal | 1 (6%) | 4 (22%) | 4 (22%) | 9 (50%) |
|  | **IgA+** 11/55 (20%) |  | **8 (73%)**^*’^ | **3 (27%)**^’^ | **0 (0%)**^^^ | **11 (100%)** |
|  |  | None | 5 (46%) | 1 (9%) | 0 (0%) | 6 (55%) |
|  |  | cPC only | 3 (27%) | 0 (0%) | 0 (0%) | 3 (27%) |
|  |  | cB-cells only | 0 (0%) | 2 (18%) | 0 (0%) | 2 (18%) |
|  |  | Subtotal | 3 (27%) | 2 (18%) | 0 (0%) | 5 (45%) |
|  | **IgG+** 24/55 (43%) |  | **16 (67%)**^*’^ | **6 (25%)**^^’^ | **1 (4%)**^^^ | **23 (96%)** |
|  |  | None | 14 (59%) | 2 (8%) | 0 (0%) | 16 (67%) |
|  |  | cPC only | 2 (8%) | 0 (0%) | 0 (0%) | 2 (8%) |
|  |  | cPC+cB-cells | 0 (0%) | 2 (8%) | 0 (0%) | 2 (8%) |
|  |  | cB-cells only | 0 (0%) | 2 (8%) | 1 (4%) | 3 (13%) |
|  |  | Subtotal | 2 (8%) | 4 (17%) | 1 (4%) | 7 (29%) |
|  | **Light-chain** 2/55 (4%) | cPC only | **1 (50%)** | **0 (0%)** | **0 (0%)** | **1 (50%)** |
| **SMM** (n= 12) | **IgG+ IgM+** 1/12 (8%) | cPC+cB-cells | **0 (0%)** | **1 (100%)** | **0 (0%)** | **1 (100%)** |
|  | **IgA+** 3/12 (25%) |  | **1 (33%)** | **2 (67%)** | **0 (0%)** | **3 (100%)** |
|  |  | None | 1 (33%) | 1 (33%) | 0 (0%) | 2 (67%) |
|  |  | cB-cells only | 0 (0%) | 1 (33%) | 0 (0%) | 1 (33%) |
|  |  | Subtotal | 0 (0%) | 1 (33%) | 0 (0%) | 1 (33%) |
|  | **IgG+** 7/12 (58%) |  | **5 (71%)**^*’^ | **2 (28%)**^’^ | **0 (0%)**^^^ | **7 (100%)** |
|  |  | None | 1 (14%) | 1 (14%) | 0 (0%) | 2 (28%) |
|  |  | cPC only | 4 (57%) | 0 (0%) | 0 (0%) | 4 (57%) |
|  |  | cPC+cB-cells | 0 (0%) | 1 (14%) | 0 (0%) | 1 (14%) |
|  |  | Subtotal | 4 (57%) | 1 (14%) | 0 (0%) | **5 (71%)** |
|  | **Light-chain** 1/12 (8%) | cPC only | **1 (100%)** | **0 (0%)** | **0 (0%)** | **1 (100%)** |
| **SWM**  (n= 8) | **IgM+** 8/8 (100%) |  | **0 (0%)** | **7 (88%)**^*^^ | **0 (0%)**^!^ | **7 (88%)** |
|  |  | None | 0 (0%) | 4 (50%) | 0 (0%) | 4 (50%) |
|  |  | cPC+cB-cells | 0 (0%) | 2 (25%) | 0 (0%) | 2 (25%) |
|  |  | cB-cells only | 0 (0%) | 1 (13%) | 0 (0%) | 1 (13%) |
|  |  | Subtotal | 0 (0%) | 3 (38%) | 0 (0%) | 3 (38%) |

^*^*p*<0.05 vs MGUS IgM in BM; ^’^*p*<0.05 vs SWM IgM in BM; ^^^*p*<0.05 vs cPC only in BM; ^!^*p*<0.05 vs cPC+cB-cells in BM. *p*<0.05 in IgM+ MGUS for cPC and cB-cells between BM and PB, and in IgG+ MGUS for cPC between BM and PB.

BM, bone marrow; cB-cells, clonal B cells; cPC, clonal plasma cells; MGUS, monoclonal gammopathy of undetermined significance; SMM, smoldering multiple myeloma; SWM, smoldering Waldenström’s macroglobulinemia.

**Supplementary Table 3.** Correlation between the distinct diagnostic subtypes of monoclonal gammopathies as per the current IMWG criteria and the immunophenotypic data profile regarding the presence or absence of clonal plasma cells and clonal B-lymphocytes in BM.

|  | | **Diagnosis** | | | | | | | | | | |
| --- | --- | --- | --- | --- | --- | --- | --- | --- | --- | --- | --- | --- |
| **Immunophenotypic profile** | | **MGUS**  (n= 55) | | | | **SMM**  (n= 12) | | | | **SWM**  (n= 8) | **Total** (n= 75) | |
|  | **IgH isotype** | **IgM** | **IgA** | **IgG** | **LC** | **IgG+IgM** | **IgA** | **IgG** | **LC** | **IgM** |  |  |
|  |  | **18**  **(33%)** | **11 (20%)** | **24 (43%)** | **2**  **(4%)** | **1**  **(8%)** | **3 (25%)** | **7 (58%)** | **1**  **(8%)** | **8**  **(100%)** |  |  |
| **MGUS** | **IgM** | 2  (4%) |  |  |  |  |  |  |  |  | 2  (3%) |  |
|  | **IgA** |  | 11 (20%) |  |  |  |  |  |  |  | 11 (15%) |  |
|  | **IgG** |  |  | 22 (40%) |  |  |  |  |  |  | 22 (29%) |  |
|  | **LC** |  |  |  | 1  (2%) |  |  |  |  |  | 1  (1%) |  |
| **SMM** | **IgM** |  |  |  |  |  |  |  |  | 1  (12%) | 1  (1%) |  |
|  | **IgA** |  |  |  |  |  | 3 (25%) |  |  |  | 3  (4%) |  |
|  | **IgG** |  |  |  |  |  |  | 7 (58%) |  |  | 7  (9%) |  |
|  | **LC** |  |  |  |  |  |  |  | 1  (8%) |  | 1  (1%) |  |
| **SWM** | **IgM** | 8  (15%) |  |  |  | 1  (8%) |  |  |  | 6  (75%) | 15 (20%) |  |
| **CLL-like MBL** | **IgM** | 4  (7%) |  |  |  |  |  |  |  |  | 4  (5%) |  |
|  | **IgG** |  |  | 1  (2%) |  |  |  |  |  |  | 1  (1%) |  |
| **None** |  | 4  (7%) |  | 1  (2%) | 1  (2%) |  |  |  |  | 1  (12%) | 7  (9%) |  |
| **N. of discordant**  **cases^*^** | | 16/18  (89%) | 0/11  (0%) | 2/24  (8%) | 1/2  (50%) | 1/1  (100%) | 0/3  (0%) | 0/7  (0%) | 0/1  (0%) | 2/8  (25%) | 22/75  (29%) |  |
| **Total** |  |  | 19/55  (35%) |  |  |  | 1/12  (8%) |  |  | 2/8  (25%) |  |  |

^*^Based on the presence of cPC and/or cB-lymphocytes in BM and their immunophenotypic characteristics (35).

cB-cells, clonal B-lymphocytes; CLL, chronic lymphocytic leukemia; cPC, clonal plasma cell; IMWG, International Myeloma Working group; LC, light chain; MGUS, monoclonal gammopathy of undetermined significance; MBL, monoclonal B-cell lymphocytosis; SMM, smoldering multiple myeloma; SWM, smoldering Waldenström’s macroglobulinemia.

**Supplementary Table 4**. Frequency of clonal plasma cells (cPC) and clonal B-lymphocytes (cB-cells) found in bone marrow vs blood of included subjects presenting with a serum M-component distributed according to diagnosis as per the IMWG criteria (n= 75).

| **Diagnosis** | **Type of clonal cells** | | | | ***p*-value** | **Total** |
| --- | --- | --- | --- | --- | --- | --- |
|  | **Blood status** | **BM status** | | |  |  |
|  |  | **cPC only**  (n= 34) | **cPC+cB-cells**  (n= 29) | **cB-cells only**  (n= 5) |  |  |
| **MGUS**  (n= 55) |  | **27 (49%)** | **17 (31%)** | **5 (9%)** | *<0.001* | **49 (89%)** |
|  | None | 20 (36%) | 7 (13%) | 0 (0%) |  | 27 (49%) |
|  | cPC only | 7 (13%) | 0 (0%) | 0 (0%) |  | 7 (13%) |
|  | cPC+cB-cells | 0 (0%) | 4 (7%) | 0 (0%) |  | 4 (7%) |
|  | cB-cells only | 0 (0%) | 6 (11%) | 5 (9%) |  | 11 (20%) |
|  | Subtotal | 7 (13%) | 10 (18%) | 5 (9%) |  | 22 (40%) |
| **SMM**  (n= 12) |  | **7 (58%)** | **5 (42%)** | **0 (0%)** | *0.008* | **12 (100%)** |
|  | None | 2 (17%) | 2 (17%) | 0 (0%) |  | 4 (33%) |
|  | cPC only | 5 (42%)^^^ | 0 (0%) | 0 (0%) |  | 5 (42%) |
|  | cPC+cB-cells | 0 (0%) | 2 (17%) | 0 (0%) |  | 2 (17%) |
|  | cB-cells only | 0 (0%) | 1 (8%) | 0 (0%) |  | 1 (8%) |
|  | Subtotal | 5 (42%) | 3 (25%) | 0 (0%) |  | 8 (67%) |
| **SWM**  (n= 8) |  | **0 (0%)** | **7 (88%)** | **0 (0%)** | *<0.001* | **7 (88%)** |
|  | None | 0 (0%) | 4 (50%) | 0 (0%) |  | 4 (50%) |
|  | cPC+cB-cells | 0 (0%) | 2 (25%) | 0 (0%) |  | 2 (25%) |
|  | cB-cells only | 0 (0%) | 1 (13%) | 0 (0%) |  | 1 (13%) |
|  | Subtotal | 0 (0%) | 3 (38%) | 0 (0%) |  | 3 (38%) |
|  | ***p*-value** | ***0.04*** | ***0.61*** | ***0.21*** |  | ***0.15*** |
| **Total**  (n= 75) |  | **12/34 (35%)** | **16/29 (55%)** | **5/5 (100%)** | ***0.07*** | **33/68 (49%)** |

^^^*p*<0.05 vs MGUS in blood.

BM, bone marrow; cB-cells, clonal B-lymphocytes; cPC, clonal plasma cells; IMWG, international myeloma working group; MGUS, monoclonal gammopathy of undetermined significance; SMM, smoldering multiple myeloma; SWM, smoldering Waldenström’s macroglobulinemia.
